# Hexasodium fytate exposure-response correlations in a randomized, placebo-controlled study of patients on dialysis with cardiovascular calcification

**DOI:** 10.3389/fphar.2024.1325186

**Published:** 2024-02-07

**Authors:** Joan Perelló, Joan Alberti, Juan Vicente Torres, Miguel D. Ferrer, M. Mar Perez, Firas Bassissi, Alex Gold, Paolo Raggi, Glenn M. Chertow, Carolina Salcedo

**Affiliations:** ^1^ Sanifit Therapeutics S.A., Palma, Spain; ^2^ Department of Chemistry, University of the Balearic Islands, Palma, Spain; ^3^ ADMETRA Consulting, Palma, Spain; ^4^ Optimapharm, Palma, Spain; ^5^ Department of Fundamental Biology and Health Sciences, University of the Balearic Islands, Palma, Spain; ^6^ Department of Medicine, Stanford University, Palo Alto, CA, United States; ^7^ Department of Medicine, Mazankowski Alberta Heart Institute, University of Alberta, Edmonton, AB, Canada

**Keywords:** pharmacokinetics, pharmacodynamics, hexasodium fytate, SNF472, calcification, cardiovascular

## Abstract

**Background:** Patients receiving dialysis have high cardiovascular risk in part due to extensive vascular calcification. In the CaLIPSO study, infusion of hexasodium fytate (SNF472), the hexasodium salt of inositol hexaphosphate, for 52 weeks thrice weekly during hemodialysis significantly reduced progression of coronary artery calcification (CAC). This report examines pharmacokinetic/pharmacodynamic (PK/PD) and exposure-efficacy in CaLIPSO.

**Methods:** We measured hexasodium fytate plasma concentrations (PK) by validated liquid chromatography-mass spectroscopy, and hydroxyapatite crystallization in plasma (PD) by validated spectrophotometry. Analyses included patients evaluable for PK, PD, and CAC change (per-protocol analysis). We developed a simple E_max_ model for maximum concentration (C_max_) and PD effect, and linear and non-linear E_max_ models for exposure-efficacy among individual average C_max_ and absolute and percent changes in CAC score from baseline to week 52.

**Results:** Among evaluable patients receiving placebo (*n* = 15), 300 mg (*n* = 20), or 600 mg (*n* = 20), average C_max_ across visits was not quantifiable (<0.76 μM), 15 μM, and 46 μM, respectively. These results suggest a more-than-proportional increase, without accumulation, with a C_max_ ratio of approximately 3 for the doses administered. Average inhibition of hydroxyapatite crystallization was 15%, 61%, and 75%, respectively, and similar across visits. Simple E_max_ models described 80% maximal effect at exposures >21.9 µM and a plateau in exposure-efficacy above the third quartile of C_max_ (≥32 µM).

**Conclusion:** Hexasodium fytate has exposure-dependent effects on hydroxyapatite crystallization and progression of cardiovascular calcification. Simple E_max_ models show robust relations among exposure, inhibition of hydroxyapatite crystallization, and change in CAC volume.

**Clinical Trial Registration:** https://www.clinicaltrials.gov; identifier NCT02966028.

## 1 Introduction

Patients receiving dialysis for the management of kidney failure experience exceptionally high rates of all-cause and cardiovascular mortality when compared to the general population (Foley et al., 1998; Baigent et al., 2000; Jegatheesan et al., 2018). Additionally, most patients receiving dialysis have evidence of cardiovascular calcification (Raggi et al., 2002; Bellasi et al., 2006). Medial calcification (Monckeberg’s medial sclerosis) occurs more commonly in patients on dialysis than in persons with normal or near normal kidney function and appears to be a key component of the extensive cardiovascular calcification found in these patients (Raggi et al., 2007; Lanzer et al., 2014). Medial calcification results from crystallization of calcium and phosphate into hydroxyapatite, which is then deposited in the elastin of the extracellular matrix in a process triggered by the vascular smooth muscle cells of the arterial wall (Lanzer et al., 2014).

Myo-inositol hexaphosphate (IP6) is found in foods with high fiber content. While IP6 provides natural protection against cardiovascular calcification related to aging, it is not absorbed well from dietary sources and parenteral administration is required to achieve supraphysiologic levels with a potential to prevent or attenuate progression of cardiovascular calcification (Joubert et al., 2016). Hexasodium fytate (SNF472), the hexasodium salt of IP6, is being developed as an intravenous formulation of IP6 for clinical use. In a series of animal studies, we showed that hexasodium fytate inhibits calcium-phosphate crystallization by binding to hydroxyapatite crystals selectively, with minimal chelation of free calcium and no deleterious effects on bone mineralization in dogs or in rat osteoblasts (Ferrer et al., 2018; Perello et al., 2020). These results supported further nonclinical and clinical investigation of hexasodium fytate for the prevention or treatment of cardiovascular calcification.

In a Phase 1b clinical study of patients on hemodialysis, we showed that exposure to hexasodium fytate increased slightly more than dose-proportionally for multiple doses ranging from 3 to 20 mg/kg, without evidence of significant accumulation (Salcedo et al., 2019). Hexasodium fytate significantly inhibited hydroxyapatite crystallization at all doses (70%–80% inhibition) with concentrations producing 80% maximal effect (EC_80_) at doses of 5.6 mg/kg (469 mg/patient), using an *ex vivo* pharmacodynamic (PD) plasma assay that we had previously developed and validated (Ferrer et al., 2017).

In the Phase 2b CaLIPSO study, we randomized patients receiving hemodialysis with cardiovascular calcification to receive infusions of placebo, hexasodium fytate 300 mg, or hexasodium fytate 600 mg thrice weekly during hemodialysis for 52 weeks (Raggi et al., 2020a). At these doses, which we selected for the Phase 2b study to be below (300 mg) or above (600 mg) the EC_80_ from the Phase 1b study, hexasodium fytate significantly attenuated progression of cardiovascular calcification. Among patients in the per-protocol population who completed study treatment, mean change in coronary artery calcification (CAC) volume score measured by computed tomography from baseline to week 52 was 24% in the placebo group and 8% in the hexasodium fytate combined dosing groups (*p* < 0.001), with mean changes of 10% in the 300 mg group and 6% in the 600 mg group. These results were encouraging, because CAC has been recognized as a risk-modifying marker in the guidelines from the American Heart Association and American College of Cardiology for the primary prevention of atherosclerotic cardiovascular disease. Previous publications of the results obtained in the CaLIPSO study include key efficacy and safety assessments (Raggi et al., 2020a), subgroup analyses (Raggi et al., 2020b) and effects of hexasodium phytate on bone (Bushinsky et al., 2021). However, the relation between the pharmacokinetics (PK) of hexasodium fytate and its efficacy or the suitability of circulating PD measurements to anticipate efficacy have not been yet reported. In this report, we analyze PK, PD, and efficacy results from the CaLIPSO study to determine the relations among hexasodium fytate exposure and its efficacy, measured as change in cardiovascular calcification.

## 2 Materials and methods

### 2.1 Study design

We reported the methods for the CaLIPSO study previously (Bellasi et al., 2021). In this Phase 2b study, we enrolled 274 patients receiving maintenance hemodialysis with CAC (baseline Agatston CAC score 100 to 3,500 Hounsfield units). We used non-contrast multi-detector computed tomography (MDCT) to obtain a minimum of 64 contiguous 3-mm tomographic slices from above the aortic arch to the diaphragm, at end-expiration; we conducted image analysis on slices acquired during late diastole or at the time of least motion. A central reader calculated both Agatston and calcium volume scores in the coronary arteries, aortic valve, and thoracic aorta at baseline (screening) and at week 52 (or earlier in case of termination in advance of the last visit).

We randomized eligible patients 1:1:1 centrally to receive placebo, hexasodium fytate 300 mg, or hexasodium fytate 600 mg, infused thrice weekly during hemodialysis for 52 weeks. Each infusion of study drug or placebo lasted 2.5 h (±30 min). The primary efficacy endpoint was the change in log CAC volume score from baseline to week 52. Secondary efficacy endpoints were changes in log Agatston and volume scores at the thoracic aorta and aortic valve. We reported results for efficacy and safety endpoints previously (Raggi et al., 2020a).

A subset of study sites that had a −70°C freezer and could send blood samples on dry ice to the central laboratory on the day of collection enrolled patients in this substudy of PK and PD. These sites used dialysis ports to collect blood samples at the beginning of week 1, 10, 22, and 52. Blood samples were collected twice at these visits: before treatment infusion and within 10 min before the end of infusion. Sites collected the blood samples into K_3_EDTA anticoagulant tubes, which they centrifuged at 3,500 rpm for 10 min and stored at −70°C until the tubes were shipped to the central laboratory on dry ice.

### 2.2 Pharmacokinetic and pharmacodynamic assays

For PK, we measured plasma concentrations of IP6 (hexasodium fytate free acid; molecular weight, 660 g/mol) with a validated liquid chromatography-mass spectroscopy (LC-MS/MS) method (Tur et al., 2013). For PD, we measured the *ex-vivo* hydroxyapatite crystallization in plasma samples with a validated method (Ferrer et al., 2017).

### 2.3 Statistical analysis

We used SAS^®^ Software Version 9.4 for data analysis and a threshold of *p* < 0.05 for statistical significance. We included patients in the per-protocol analysis set for analyses of change in CAC if they met all inclusion and exclusion criteria, received 80% or more of scheduled treatment, completed the study and the week 52 dosing visit, and had evaluable MDCT scans at baseline and within 120 days after the week 52 dosing visit. We included patients in the PK/PD per-protocol analysis set if they had at least one measurable hexasodium fytate concentration and one measurable PD at any visit (week 1, 10, 22, or 52) that was obtained within the 10-min window before the end of infusion.

We assessed accumulation effects after repeated dosing by comparing maximum concentration (C_max_) between visits. We designated accumulation to have developed if the 95% confidence interval (CI) of the difference did not include 0. Due to the small number of patients with evaluable samples at week 52, we used a linear model to analyze the visit effect on C_max_. In addition, we conducted an individual evaluation of accumulation for the 600 mg group.

We analyzed the effect of visit (week), treatment group, and the interaction between visit and treatment group on the percentage of PD inhibition values following the same methods described above for C_max_.

In the PK/PD analysis, we examined the relation between average C_max_ (from end of infusion samples) and average PD effect (percentage of hydroxyapatite inhibition in plasma samples across 52 weeks of treatment). In the exposure-efficacy analysis, we examined the relation between average C_max_ (from end of infusion samples) and percent CAC volume change from baseline. We used individual values for each patient to construct a linear mixed model evaluating the effect of visit (week), treatment group, and the interaction between visit and treatment group on C_max_ while considering an unstructured correlation between repeated measures within a patient. We conducted estimates of the parameters associated with these two factors for the prediction of C_max_, with their corresponding standard error, 90% confidence intervals, and *p*-values.

We assessed the PK/PD relations using the nonlinear E_max_ model, which is particularly effective when the effect of a drug intensifies at higher concentrations and then reaches a plateau. The nonlinear E_max_ model is parametrized as follows:

$${PD}_{i}=E_{0}+\frac{{C_{\max}}_{i}^{H}*\;E_{\max}}{{C_{\max}}_{i}^{H}+{{EC}_{50}}^{H}}+\varepsilon_{i}$$

Where:

i: patient indicator.

PD: value of the response.

C_max_: maximum plasma concentration.

E_0_: estimated minimal effect.

E_max_: estimated maximal net effect (attributable to the drug).

EC_50_: concentration of drug producing 50% of E_max_.

H: slope factor (Hill factor), measuring sensitivity of the response to the dose range of the drug, determining the steepness of the dose-response curve (H > 0).

ε: random error term; ε_i_ terms for individual patients were assumed to be independent and identically (and normally) distributed, with a mean of 0 and variance σ^2^.

We used a nonlinear mixed effects model to estimate fixed effects parameters (E_max_, E_0_, H, EC_50_) and interindividual random effects parameters (including week as covariate). We assumed that the interindividual variability of structural model parameters (fixed effects parameters) followed a normal distribution, using an additive error model as follows:

$$P_{i}=TVP+\eta_{i}$$

where P_i_ was the predicted parameter value for patient i, TVP was the typical population value and n_i_ ∼ N (0, ω^2^) was a random variable representing the difference between individual values and the typical value of the parameter. We excluded from the analysis data points where the absolute value of studentized residuals were 3 or more during the initial model building process.

We selected the best model with the highest adjusted coefficient of determination (R^2^). We examined predicted versus observed values for goodness of fit.

When examining the relation between exposure (average C_max_) and efficacy (reduction of CAC progression), we explored simple (assuming a Hill slope of H = 1) and sigmoid (H as a variable) E_max_ models. The linear model used to evaluate the relations between efficacy and exposure has the following form:

$$Y=b0+b1X+\varepsilon_{i}$$

Where:

Y: value of the response.

b0: Y-axis intercept.

b1X: parameter estimate (slope) for variable X.

ε_i_: random error term; ε_i_ terms for individual patients were assumed to be independent and identically (and normally) distributed, with a mean of 0 and variance σ^2^.

We summarized goodness of fit for the linear model as described above for P_i_.

When plasma concentrations were below the lower limit of quantification (LQ) of 0.76 μM, we considered them to be equal to LQ/2 for descriptive analysis and modeling purposes. In addition, we conducted a sensitivity analysis for the PK/PD modeling results considering values below the LQ as equal to 0 and a second sensitivity analysis considering them as equal to the LQ.

We identified outlier values as those PK or PD observations that were more than 3 intraindividual standard deviations apart from the median of the subject population. We excluded missing PK and PD values from descriptive statistics and modeling.

### 2.4 Study approval

Patients gave written informed consent to participate. We obtained ethics approval from an institutional review board for each study site, in accordance with the local/national processes. We conducted the study in accordance with the Declaration of Helsinki, International Council for Harmonisation Guidelines on Good Clinical Practice, and regulatory requirements. The clinical trial was registered at ClinicalTrials.gov as # NCT02966028.

## 3 Results

### 3.1 Analysis sets

**FIGURE 1:**
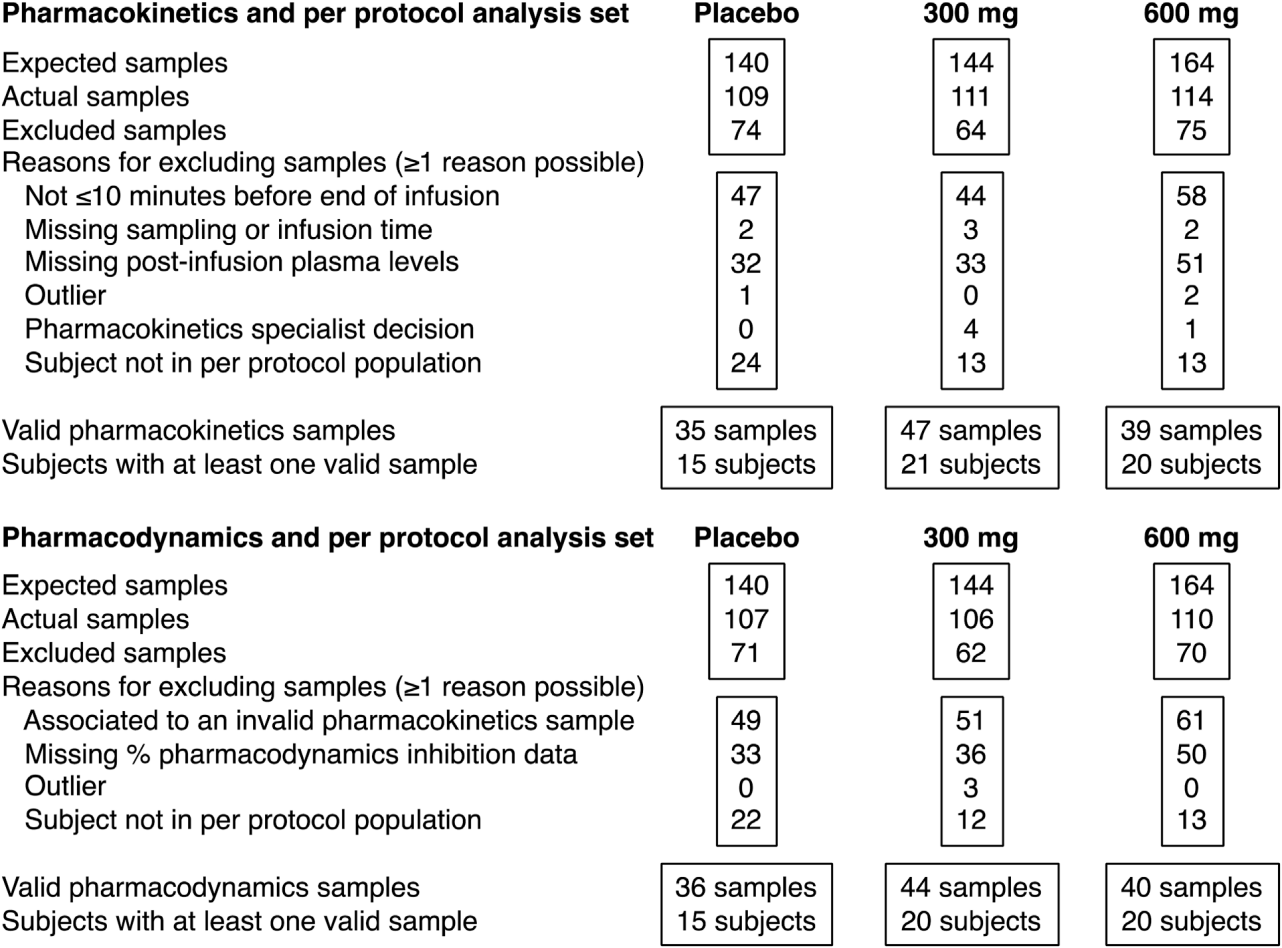
Patient disposition and analysis sets.

At the study sites that participated in PK and PD assessments, 111 patients provided at least one evaluable blood sample. A total of 55 patients (15 in the placebo group, 20 in the hexasodium fytate 300 mg group, and 20 in the hexasodium fytate 600 mg dosing group) met the criteria for per-protocol analyses of both CAC and PK/PD (Figure 1). A total of 116 plasma samples from these patients were evaluable for both PK and PD across visits at week 1, 10, 22, and 52 (Table 1).

**TABLE 1:** Number of patients providing blood samples for both pharmacokinetic and pharmacodynamic analyses at the start and end of infusion (within the last 10 minutes) by treatment and visit.

Treatment week	Placebo (*n* = 15)	300 mg (*n* = 20)	600 mg (*n* = 20)	Total (*n* = 55)
Week 1	8 (53.3)	11 (55.0)	9 (45.0)	28 (50.9)
Week 10	9 (60.0)	12 (60.0)	9 (45.0)	30 (54.5)
Week 22	9 (60.0)	11 (55.0)	15 (75.0)	35 (63.6)
Week 52	8 (53.3)	10 (50.0)	6 (30.0)	23 (41.8)

Data are shown as n (%).

### 3.2 Pharmacokinetic profile

**FIGURE 2:**
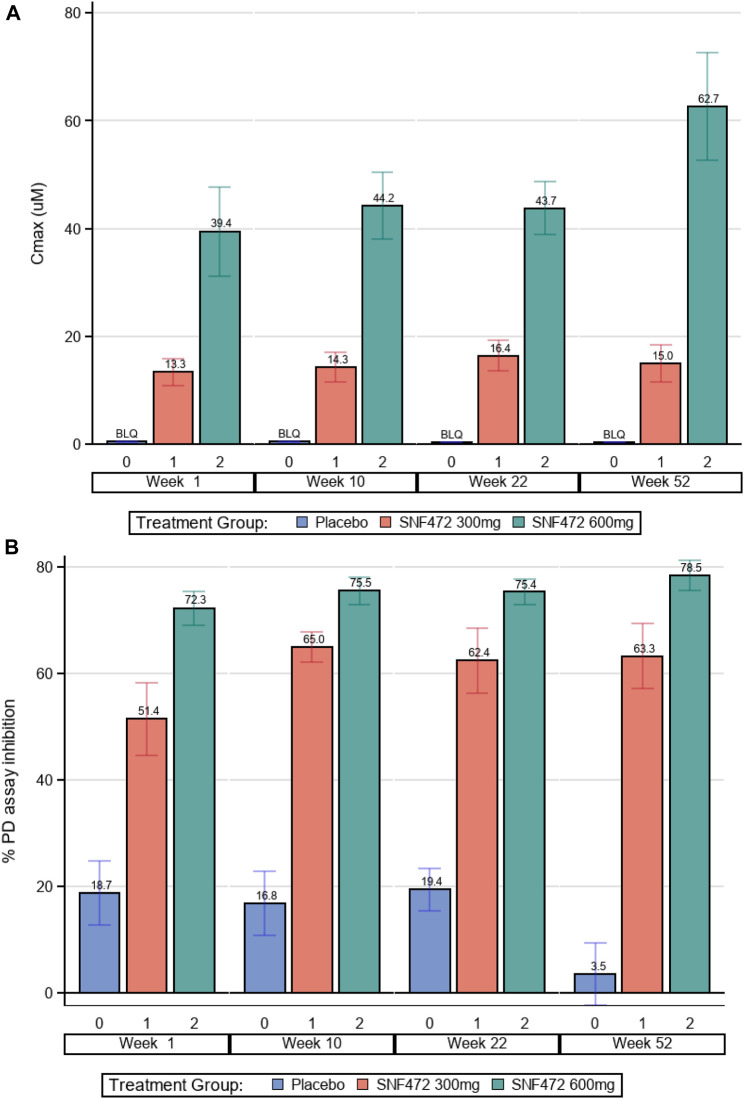
Pharmacokinetics and Pharmacodynamics. **(A)** Hexasodium Fytate Maximum Plasma Concentration (C_max_). Average C_max_ was significantly different between each hexasodium fytate group and placebo (*p* < 0.001); C_max_ values by visit were not significantly different within each treatment group (*p* = 0.631). **(B)** Mean Inhibition of Calcium-Phosphate (Hydroxyapatite) Crystallization. The average PD effect was significantly different between each hexasodium fytate treatment group and placebo (*p* < 0.001); the PD effect by visit was not significantly different within each treatment group (*p* = 0.652).

Hexasodium fytate plasma concentrations, measured as free acid IP6, were determined in all plasma samples at the end of infusion. In the placebo group, mean plasma C_max_ for hexasodium fytate was not quantifiable (below 0.76 μM) at each assessment (Table 2; Figure 2A). In the 300 mg group, mean plasma C_max_ was approximately 15 μM, averaged across visits and at each assessment. In the 600 mg group, mean plasma C_max_ averaged 46 µM across visits, with mean values of approximately 40–45 μM at week 1, 10, and 22, and approximately 60 μM at week 52. This increase at week 52 appeared to be an artifactual result because concentrations were available for only six patients. Further evaluation showed that most of these patients already had high hexasodium fytate concentrations since the beginning of the study, and individual assessment showed that there was no evidence of accumulation throughout the study, with a mean (SD) accumulation ratio for C_max_ of 1.19 (0.23) at week 52 for the 600 mg dose (data on file). The C_max_ was significantly different between each hexasodium fytate group and placebo (*p* < 0.001) but was not significantly different by visit within each treatment group (*p* = 0.63). The mean C_max_ ratio between the 600 mg and 300 mg groups was 3.0, which suggests saturation of hexasodium fytate clearance after intravenous administration.

**TABLE 2:** Mean (SD) C_max_ and percent of hydroxyapatite crystallization by treatment and visit.

Treatment	Value	Week 1	Week 10	Week 22	Week 52	Average
C_max_, µM						
Placebo	n	8	9	10	8	35
	Mean (SD)	BLQ	BLQ	BLQ	BLQ	BLQ
300 mg	n	13	12	11	11	47
	Mean (SD)	13.3 (9.1)	14.3 (9.6)	16.4 (9.3)	15.0 (11.5)	14.7 (9.6)
600 mg	n	9	9	15	6	39
	Mean (SD)	39.4 (24.7)	44.2 (18.7)	43.7 (19.1)	62.7 (24.3)	45.8 (21.7)
PD effect, %						
Placebo	n	9	10	9	8	36
	Mean (SD)	18.7 (18.2)	16.8 (18.8)	19.4 (11.9)	3.5 (16.5)	15.0 (17.1)
300 mg	n	11	12	11	10	44
	Mean (SD)	51.4 (22.4)	65.0 (10.0)	62.4 (20.4)	63.3 (19.2)	60.6 (18.6)
600 mg	n	9	9	15	7	40
	Mean (SD)	72.3 (9.6)	75.5 (7.8)	75.4 (9.4)	78.5 (7.7)	75.2 (8.7)

n, number of samples.

BLQ, below limit of quantification (0.76 µM).

Average, arithmetic mean of all values obtained in week 1, 10, 22, and 52.

PD effect, pharmacodynamic effect, measured as inhibition of hydroxyapatite crystallization.

### 3.3 Pharmacodynamic profile

Using a validated assay to measure the PD effect of hexasodium fytate treatment, average inhibition of hydroxyapatite crystallization in the placebo, 300 mg, and 600 mg dose groups was 15%, 61%, and 75%, respectively (Table 2). The PD effect was significantly different between each hexasodium fytate treatment group and placebo (*p* < 0.001) but was not significantly different by visit within each treatment group (*p* = 0.65) (Table 2; Figure 2B). The mean PD effects in the 300 mg and 600 mg groups compared with the placebo group were increased 3.9-fold and 4.6-fold, respectively.

### 3.4 Pharmacokinetic-pharmacodynamic relation

**FIGURE 3:**
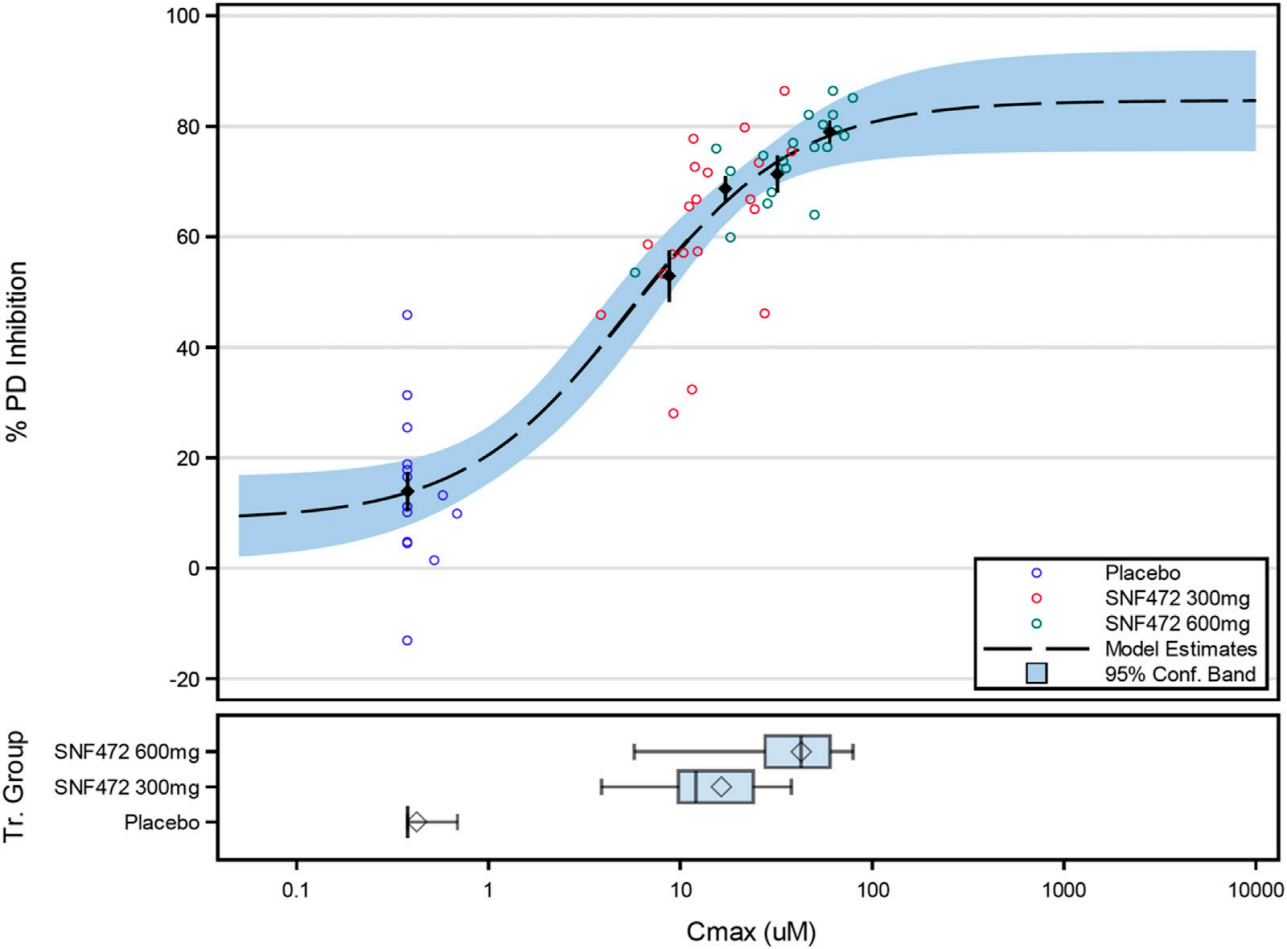
Final PK/PD Model for the Relation Between Average Hexasodium Fytate Maximum Concentration (C_max_) and Average Inhibition of Hydroxyapatite Crystallization (% PD inhibition). Black points and lines: mean (±SE) for % PD inhibition by C_max_ quartile. Box plots for C_max_: diamond represents the arithmetic mean, vertical line represents the median, central rectangle spans the first quartile to the third quartile (the interquartile range), and whiskers show the locations of the minimum and maximum.

A sigmoidal E_max_ model fit the PK and PD data well (Figure 3). Assuming a Hill coefficient of H = 1, model fitting improved from R^2^ = 0.8370 (simple E_max_ model) to R^2^ = 0.8400 (Hill E_max_ model). By setting values below the LQ to be equal to LQ/2, the adjusted net E_max_ for inhibition of hydroxyapatite crystallization was 76% and the E_0_ was 9% (Table 3). A hexasodium fytate plasma concentration of 5.5 µM was the concentration associated with half-maximal effect (EC_50_) for inhibition of hydroxyapatite crystallization and a plasma concentration of 21.9 µM was the EC_80_. Sensitivity analyses for the PK/PD model showed that setting values below the LQ to be equal to 0, LQ/2, or LQ yielded similar results, with EC_50_ values of 6.3, 5.5, and 4.7 µM, respectively (Table 4).

**TABLE 3:** Final PK/PD model using average C_max_ and average inhibition of hydroxyapatite crystallization.

Source	DF	Sum of squares	Mean square	F-value	*p*-value
Observations used	55				
Model	2	35,283	17,641	139.6	<0.001
Error	52	6,574	126.4	–	–
Corrected	Total	41,856	–	–	–
Parameter	Estimate	SE	95% CI	t-value	*p*-value
E_max_, % inhibition	75.9	4.81	66.3, 85.6	15.78	<0.001
E_0_, % inhibition	8.78	3.82	1.13, 16.4	2.30	0.025
EC_50_, µM	5.48	1.76	1.95, 9.01	3.11	0.003
EC_80_, µM	21.9	–	7.79, 36.0	–	–

DF, degrees of freedom; E_max_, estimated maximum effect; E_0_, estimated minimum effect; EC_50_, hexasodium fytate concentration associated with 50% maximum effect; EC_80_, hexasodium fytate concentration associated with 80% maximum effect.

Values below the limit of quantification (LQ; 0.758 µM) were set to LQ/2 (0.379 µM).

**TABLE 4:** Influence of the assumed value for observed values below the limit of quantification in the final PK/PD model.

Model	E_max_, % inhibition	E_0_, % inhibition	EC_50_, µM	R^2^
BLQ = 0				
Estimate	72.0	13.5	6.33	0.8418
% RSE	7.36	22.2	29.9	
95% CI	61.4, 82.6	7.48, 19.5	2.54, 10.1	
BLQ = LQ/2				
Estimate	75.9	8.78	5.48	0.8430
% RSE	6.34	43.44	32.11	
95% CI	66.3, 85.6	1.13, 16.4	1.95, 9.01	
BLQ = LQ				
Estimate	81.4	2.59	4.66	0.8439
% RSE	6.02	211.9	35.1	
95% CI	71.5, 91.2	−8.43, 13.6	1.38, 7.93	

BLQ, below the limit of quantification; E_max_, estimated maximum effect; E_0_, estimated minimum effect; EC_50_, hexasodium fytate concentration associated with 50% maximum effect; LQ, limit of quantification (0.758 µM); %RSE, 100*standard error/estimate.

### 3.5 Exposure-efficacy

**FIGURE 4:**
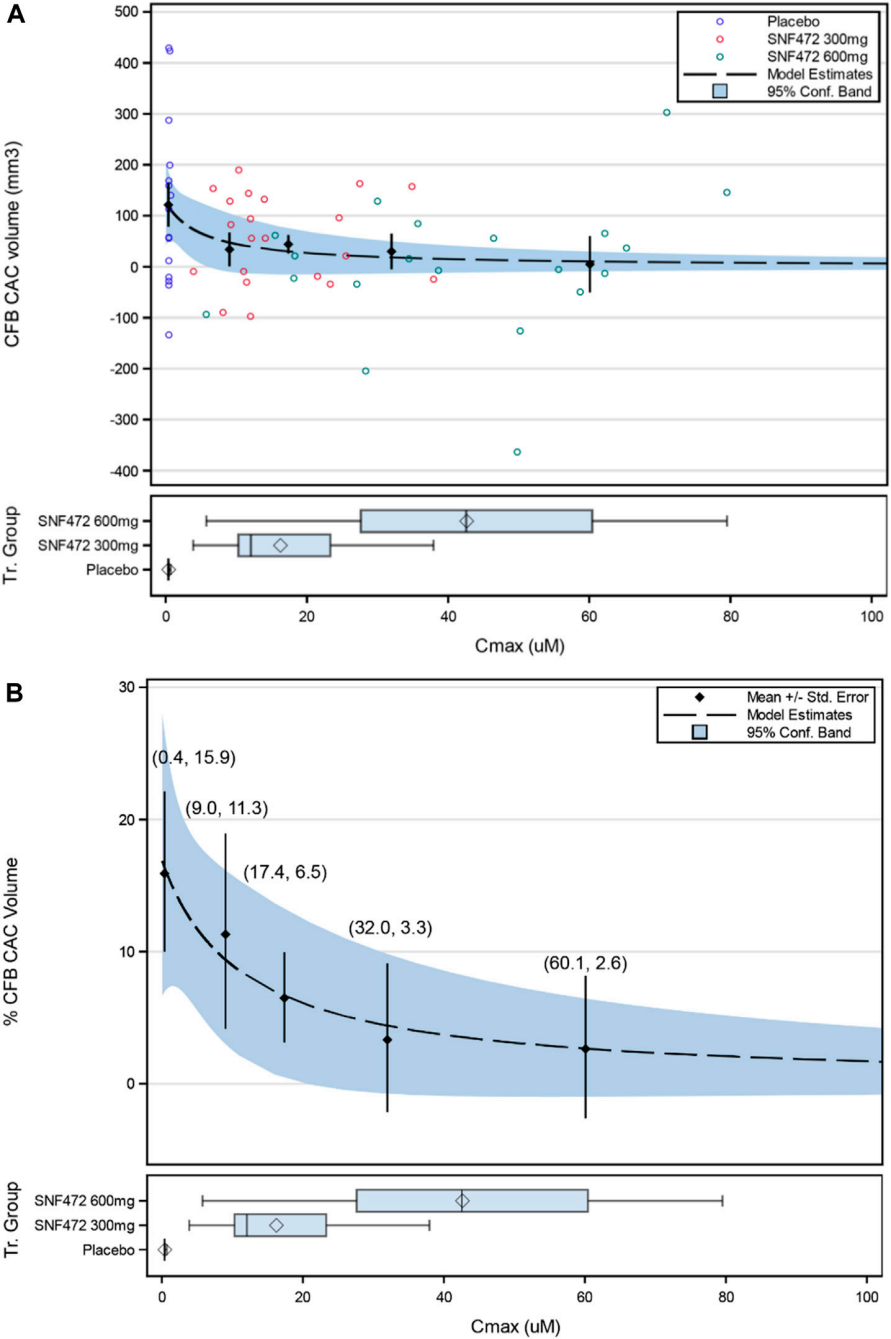
Simple E_max_ Model for the Relation of Average C_max_ Over 52 Weeks on Percentage Change from Baseline in Coronary Artery Calcium (CFB CAC) Volume (Back-transformed Values): **(A)** Individual Values; **(B)** Mean Values (±SE). Values in parentheses are (quartile average C_max_, %CFB CAC). Box Plot display: diamond represents the arithmetic mean, vertical line represents the median, central rectangle spans the first quartile to the third quartile (the interquartile range), and whiskers show the locations of the minimum and maximum.

A simple E_max_ model for the relation between exposure (average C_max_) and change from baseline of log CAC volume provided a good fit to the experimental data (Figure 4). The best fit was obtained by adjusting by baseline log CAC volume and assuming E_max_ at week 52 was the negative of that observed with placebo (E_max_ = –E_0_), which provided the highest adjusted R^2^ of 0.1857. By using this model, E_0_ represents the percent progression of CAC score in the absence of treatment and the E_max_ represents the maximum percent progression of CAC that can be attenuated by the treatment with hexasodium fytate. This model provided estimated values for back-transformed E_0_ of 16.9% (95% CI: 6.6%, 28.2%) and E_max_ of −14.5% (95% CI: −22.0%, −6.2%) (Table 5), translating into 16.9% CAC progression in the placebo group projected by the model and 2.4% CAC progression as maximal hexasodium fytate effect. The EC_50_ for CAC volume progression was 12.2 µM (95% CI: −12.1, 36.5), and the EC_80_ was 48.7 µM (95% CI: −48.4, 145.9). Based on the simple E_max_ model, there was little difference between reduction of CAC volume progression between the third quartile (32.0 µM) and fourth quartile (60.1 µM) for C_max_, with predicted CAC progression rates of 3.3% and 2.6%, respectively (Figure 4).

**TABLE 5:** Final simple model for the relation between average C_max_ over 52 Weeks and percentage change from baseline in coronary artery calcium volume score.

Source	DF	Sum of squares	Mean squares	F-value	*p*-value
Model	3	0.7	0.2	9.10	<0.001
Error	53	1.4	0.0	—	—
Uncorrected total	56	2.2	—	—	—
Parameter	Estimate	SE	95% CI	t-value	*p*-value
E_max_, % CAC change	−14.5	4.7	−22.0, −6.19	−3.39	0.001
E_0_, % CAC change	16.9	4.7	6.6, 28.2	−3.39	0.001
EC_50,_ µM	12.2	12.1	−12.1, 36.5	1.01	0.319
EC_80_, µM	48.7	—	−48.4, 145.9	—	—

CAC, coronary artery calcium volume; DF, degrees of freedom; E_max_, estimated maximum effect; E_0_, estimated minimum effect; EC_50_, hexasodium fytate concentration associated with 50% maximum effect; EC_80_, hexasodium fytate concentration associated with 80% maximum effect. In this model, E_0_ represents the percent progression of CAC, score in the absence of treatment and the E_max_ represents the maximum percent progression of CAC, that can be attenuated by the treatment with hexasodium fytate.

Values below the limit of quantification (LQ) were set to LQ/2 (0.379 µM).

## 4 Discussion

The randomized, placebo-controlled CaLIPSO study showed that adding hexasodium fytate 300 mg or 600 mg by intravenous infusion in the dialysis line during each hemodialysis session, thrice weekly for 52 weeks, significantly reduced progression of CAC (Raggi et al., 2020a). In the current analysis, we examined PK and PD results from a subset of patients in CaLIPSO who provided plasma samples at baseline and the end of hexasodium fytate infusion and established the relations among PK, PD, and therapeutic efficacy. Plasma C_max_ (measured as IP6, the free acid of hexasodium fytate) was not quantifiable in the placebo group, compared with approximately 15 µM in the 300 mg group and 45 µM in the 600 mg group. There was no evidence of hexasodium fytate accumulation over 52 weeks of treatment, which was consistent with results of previous studies of patients receiving hemodialysis treated with hexasodium fytate for 4 weeks (Salcedo et al., 2019) or for 12 weeks (Brandenburg et al., 2019). In this 52-week study, the mean C_max_ ratio between the 600 mg and 300 mg groups was approximately 3, which suggests non-proportionality that may be explained by a saturation of hexasodium fytate clearance at the highest dose.

A simple E_max_ model fit the observed PK and PD data well and showed that plasma concentrations of hexasodium fytate greater than 5 µM are associated with half-maximal response and a concentration of 21.9 µM is the EC_80_. The observed mean value for C_max_ approached this threshold in the 300 mg group (∼15 µM) and was well above it in the 600 mg group (∼45 µM).

A simple E_max_ model also fit the exposure-efficacy relations for hexasodium fytate C_max_ and the primary clinical endpoint of percent change in CAC volume score from baseline to week 52. The EC_50_ and EC_80_ values for reduction of CAC progression were hexasodium fytate concentrations of 12.2 µM and 48.7 µM, respectively, which were close to the observed mean values for C_max_ in the 300 mg and 600 mg groups, respectively. The model showed a plateau in the reduction of CAC volume progression from the third quartile (∼32 µM) for C_max_, providing further evidence that most patients receiving hexasodium fytate at a dose of 600 mg achieved an exposure within the range required for maximal therapeutic benefit.

The model predicted a maximum progression of 16.9% in CAC without exposure to hexasodium fytate, which approximated the values observed in the placebo group in the primary efficacy analysis: 20% progression in the modified intention-to-treat population and 24% in the per-protocol population (Raggi et al., 2020a). The model also predicted a maximal effect of −14.5% CAC progression between placebo and optimal hexasodium fytate exposure (16.9% progression with placebo vs. 2.4% at optimal exposures to hexasodium fytate). The actual mean changes for CAC progression from baseline to week 52 for the 600 mg hexasodium fytate dosing group in the primary analysis were 10% in the modified intention-to-treat population and 6% in the per-protocol population (Raggi et al., 2020a), suggesting good model fit.

The PD effect of hexasodium fytate in CaLIPSO, which we measured with a previously validated assay for inhibition of hydroxyapatite crystallization (Ferrer et al., 2017), showed average values of 15%, 61%, and 75% in the placebo, 300 mg, and 600 mg groups, respectively. Values below 20% are considered background noise for the PD assay (Perelló et al., 2004; Ferrer et al., 2017; Salcedo et al., 2019). The average inhibition of hydroxyapatite for the 600 mg group (75%) was similar to the E_max_ estimated by the model (76%), and also compatible with previous clinical (Salcedo et al., 2019) and nonclinical data (Ferrer et al., 2017). Average inhibition of hydroxyapatite for the 300 mg group produced a suboptimal effect, below the EC_80_, as was expected according to clinical data (Salcedo et al., 2019). The observed exposure to hexasodium fytate and observed PD effects of hexasodium fytate were consistent from the first to last week of the 52-week study, with no significant difference across visits.

A limitation of this analysis was the relatively low number of patients at the end of the study with evaluable samples, including only six patients in the hexasodium fytate 600 mg group at week 52. A subset of study sites participated in blood sample collection for PK and PD and study sites had a short window to collect blood samples for C_max_ (within the last 10 min of infusion). Failure to adhere to this timeframe could result in outlier values that were not included in the analysis. Moreover, it would have been impractical to keep patients beyond completion of their hemodialysis sessions as other patients were waiting to begin their own. However, the final PK/PD and exposure-response analysis included 116 samples for PK/PD measurements across all visits and the number of participants with any evaluable PK/PD samples was evenly distributed across the treatment groups. The consistency of the observed effects across week 1, 10, 22, and 52 of this study, as well as across individual patients within each dosing group, suggests that a larger population would have similar PK/PD and exposure-efficacy results.

## 5 Conclusion

In conclusion, simple E_max_ models for PK, PD, and efficacy data from the CaLIPSO study showed robust relations among hexasodium fytate plasma concentrations and inhibition of hydroxyapatite crystallization and CAC progression with 52 weeks of hexasodium fytate treatment in patients receiving maintenance hemodialysis. Higher hexasodium fytate exposure correlated with larger inhibition of hydroxyapatite crystallization and reduction in CAC progression. Exposure to hexasodium fytate in the 300 mg group was close but below the threshold for maximal PD effect and maximal clinical benefit, and exposure in the 600 mg group was consistently above these thresholds.

## Data Availability

The datasets presented in this article are not readily available because proprietary restrictions apply to the dataset. Requests to access the datasets should be directed to CS, carolina.salcedo@sanifit.com.

## References

[B1] Baigent C. Burbury K. Wheeler D. (2000). Premature cardiovascular disease in chronic renal failure. Lancet 356 (9224), 147–152. 10.1016/S0140-6736(00)02456-9 10963260

[B2] Bellasi A. Ferramosca E. Muntner P. Ratti C. Wildman R. P. Block G. A. (2006). Correlation of simple imaging tests and coronary artery calcium measured by computed tomography in hemodialysis patients. Kidney Int. 70 (9), 1623–1628. 10.1038/sj.ki.5001820 16955104

[B3] Bellasi A. Raggi P. Bover J. Bushinsky D. A. Chertow G. M. Ketteler M. (2021). Trial design and baseline characteristics of CaLIPSO: a randomized, double-blind placebo-controlled trial of SNF472 in patients receiving haemodialysis with cardiovascular calcification. Clin. Kidney J. 14 (1), 366–374. 10.1093/ckj/sfz144 33564440 PMC7857813

[B4] Brandenburg V. M. Sinha S. Torregrosa J. V. Garg R. Miller S. Canals A. Z. (2019). Improvement in wound healing, pain, and quality of life after 12 weeks of SNF472 treatment: a phase 2 open-label study of patients with calciphylaxis. J. Nephrol. 32 (5), 811–821. 10.1007/s40620-019-00631-0 31401795

[B5] Bushinsky D. A. Raggi P. Bover J. Ketteler M. Bellasi A. Rodriguez M. (2021). Effects of myo-inositol hexaphosphate (SNF472) on bone mineral density in patients receiving hemodialysis: an analysis of the randomized, placebo-controlled CaLIPSO study. Clin. J. Am. Soc. Nephrol. 16 (5), 736–745. 10.2215/CJN.16931020 33835939 PMC8259477

[B6] Ferrer M. D. Ketteler M. Tur F. Tur E. Isern B. Salcedo C. (2018). Characterization of SNF472 pharmacokinetics and efficacy in uremic and non-uremic rats models of cardiovascular calcification. PLoS ONE 13 (5), e0197061. 10.1371/journal.pone.0197061 29742152 PMC5942814

[B7] Ferrer M. D. Perez M. M. Canaves M. M. Buades J. M. Salcedo C. Perello J. (2017). A novel pharmacodynamic assay to evaluate the effects of crystallization inhibitors on calcium phosphate crystallization in human plasma. Sci. Rep. 7 (1), 6858. 10.1038/s41598-017-07203-x 28761091 PMC5537272

[B8] Foley R. N. Parfrey P. S. Sarnak M. J. (1998). Clinical epidemiology of cardiovascular disease in chronic renal disease. Am. J. Kidney Dis. 32 (5 Suppl. 3), S112–S119. 10.1053/ajkd.1998.v32.pm9820470 9820470

[B9] Jegatheesan D. Cho Y. Johnson D. W. (2018). Clinical studies of interventions to mitigate cardiovascular risk in peritoneal dialysis patients. Semin. Nephrol. 38 (3), 277–290. 10.1016/j.semnephrol.2018.02.007 29753403

[B10] Joubert P. Ketteler M. Salcedo C. Perello J. (2016). Hypothesis: phytate is an important unrecognised nutrient and potential intravenous drug for preventing vascular calcification. Med. Hypotheses 94, 89–92. 10.1016/j.mehy.2016.07.005 27515210

[B11] Lanzer P. Boehm M. Sorribas V. Thiriet M. Janzen J. Zeller T. (2014). Medial vascular calcification revisited: review and perspectives. Eur. Heart J. 35 (23), 1515–1525. 10.1093/eurheartj/ehu163 24740885 PMC4072893

[B12] Perello J. Ferrer M. D. Del Mar Perez M. Kaesler N. Brandenburg V. M. Behets G. J. (2020). Mechanism of action of SNF472, a novel calcification inhibitor to treat vascular calcification and calciphylaxis. Br. J. Pharmacol. 177 (19), 4400–4415. 10.1111/bph.15163 32557649 PMC7484563

[B13] Perelló J. Isern B. Muñoz J. A. Valiente M. Grases F. (2004). Determination of phytate in urine by high-performance liquid chromatography–mass spectrometry. Chromatographia 60 (5-6), 265–268. 10.1365/s10337-004-0379-5

[B14] Raggi P. Bellasi A. Bushinsky D. Bover J. Rodriguez M. Ketteler M. (2020a). Slowing progression of cardiovascular calcification with SNF472 in patients on hemodialysis: results of a randomized phase 2b study. Circulation 141 (9), 728–739. 10.1161/CIRCULATIONAHA.119.044195 31707860

[B15] Raggi P. Bellasi A. Sinha S. Bover J. Rodriguez M. Ketteler M. (2020b). Effects of SNF472, a novel inhibitor of hydroxyapatite crystallization in patients receiving hemodialysis - subgroup analyses of the CALIPSO trial. Kidney Int. Rep. 5 (12), 2178–2182. 10.1016/j.ekir.2020.09.032 33305110 PMC7710828

[B16] Raggi P. Boulay A. Chasan-Taber S. Amin N. Dillon M. Burke S. K. (2002). Cardiac calcification in adult hemodialysis patients. A link between end-stage renal disease and cardiovascular disease? J. Am. Coll. Cardiol. 39 (4), 695–701. 10.1016/s0735-1097(01)01781-8 11849871

[B17] Raggi P. Giachelli C. Bellasi A. (2007). Interaction of vascular and bone disease in patients with normal renal function and patients undergoing dialysis. Nat. Clin. Pract. Cardiovasc Med. 4 (1), 26–33. 10.1038/ncpcardio0725 17180147

[B18] Salcedo C. Joubert P. H. Ferrer M. D. Canals A. Z. Maduell F. Torregrosa V. (2019). A phase 1b randomized, placebo-controlled clinical trial with SNF472 in haemodialysis patients. Br. J. Clin. Pharmacol. 85 (4), 796–806. 10.1111/bcp.13863 30632182 PMC6422667

[B19] Tur F. Tur E. Lentheric I. Mendoza P. Encabo M. Isern B. (2013). Validation of an LC-MS bioanalytical method for quantification of phytate levels in rat, dog and human plasma. J. Chromatogr. B Anal. Technol. Biomed. Life Sci. 928, 146–154. 10.1016/j.jchromb.2013.03.023 23639799

